# Supplementary material for: Relationship between Cardiovascular Risk Factors and Composite Cardiovascular Outcomes in Patients Hospitalized with Takotsubo Syndrome: A Nationwide Analysis
Source: Med Sci (Basel). 2023 Sep 21;11(3):62. doi: 10.3390/medsci11030062 (PMC10536314; doi:10.3390/medsci11030062)
Supplement: Supplementary file 1 [file medsci-11-00062-s001.zip › medsci-2589175-supplementary.pdf]

Supplementary Materials: Relationship between Cardiovascular Risk Factors and Composite Cardiovascular Outcomes in Patients Hospitalized with Takotsubo Syndrome: A Nationwide Analysis

Nanush Damarlapally, Rupak Desai, Aanchal Sawhney, Jyoti Verma, Harroop Singh Klair, Dhanush Kolli, Birimroz Singh Sibia, Vardhan Chalasani, Rasya Reddy, Jithin Kolli, Ikechukwu Ogbu and Jyotsna Gummadi

Table S1. Modifiable CVD Risk Factors as Predictors of Cardiovascular Outcomes in Takotsubo Syndrome-related Hospitalizations.

	**All-cause mortality**				**Cardiogenic Shock**				**Dysrhythmia**				**Stroke**				**Acute VTE**			
**Comorbidity as predictor**	**OR**	**95% CI**		**P**	**OR**	**95% CI**		**P**	**OR**	**95% CI**		**P**	**OR**	**95% CI**		**P**	**OR**	**95% CI**		**P**
Hypertension, complicated	1.01	0.81	1.26	0.924	1.13	0.91	1.41	0.255	**1.23**	**1.08**	**1.40**	**0.001**	**1.34**	**1.04**	**1.73**	**0.025**	**0.73**	**0.54**	**0.98**	0.037
Diabetes with chronic complications	**1.44**	**1.12**	**1.86**	**0.005**	1.16	0.90	1.49	0.247	1.08	0.92	1.26	0.360	1.12	0.84	1.50	0.421	0.94	0.66	1.35	0.747
Diabetes without chronic complications	0.82	0.57	1.19	0.304	**0.65**	**0.44**	**0.96**	**0.032**	0.92	0.77	1.11	0.403	0.79	0.54	1.15	0.214	0.90	0.56	1.46	0.677
Hyperlipidemia	**0.54**	**0.44**	**0.67**	**<0.001**	**0.72**	**0.60**	**0.86**	**<0.001**	**0.87**	**0.78**	**0.97**	**0.012**	**0.76**	**0.62**	**0.93**	**0.007**	**0.70**	**0.54**	**0.90**	0.005
Obesity	1.21	0.88	1.66	0.245	1.15	0.88	1.50	0.297	**1.21**	**1.04**	**1.42**	**0.016**	1.00	0.72	1.38	0.991	**1.61**	**1.16**	**2.23**	0.005
Tobacco use disorder	0.83	0.63	1.09	0.173	0.86	0.67	1.11	0.246	**0.85**	**0.74**	**0.98**	**0.028**	1.14	0.87	1.48	0.334	0.77	0.54	1.10	0.157

OR = odds ratio, 95% CI= 95% confidence interval, p<0.05 indicate statistical significance. Covariates adjusted for multivariable regression analysis included age at admission, sex, race, patient's median household income quartile based on ZIP Code, admission type (elective or non-elective), primary expected payer, hospital location/teaching status, hospital region, and comorbid conditions including alcohol abuse, depression, peripheral vascular disease, prior myocardial infarction, chronic pulmonary disease, anxiety and fear-related disorders, cancer, prior transient ischemic attack or stroke without neurologic deficit, prior venous thromboembolism, complicated and uncomplicated hypertension, diabetes with and without chronic complications, hyperlipidemia, obesity, and tobacco use disorder.
